# Sex-specific differences in serum phosphate persist in patients with CKD

**DOI:** 10.1590/2175-8239-JBN-2025-0345en

**Published:** 2026-05-11

**Authors:** Martina Schatz, Rafael Tavares, Luciene M dos Reis, Rosilene M. Elias, Rosa M. A. Moyses

**Affiliations:** 1 University of Edinburgh Medical School, Edinburgh, United Kingdom.; 2 Universidade de São Paulo, Faculdade de Medicina, Hospital das Clínicas, Serviço de Nefrologia, SP, Brazil.; 3 Universidade de São Paulo, Faculdade de Medicina, LIM 16 do Hospital das Clínicas, SP, Brazil.

**Keywords:** Renal Insufficiency, Chronic, Chronic Kidney Disease–Mineral and Bone Disorder, Phosphate

## Abstract

**Introduction:**

Phosphate plays a critical role in numerous metabolic processes, and sex-specific differences have been identified in its concentrations in the general population. However, such differences have not been investigated in the context of CKD, in which additional factors may exert a more pronounced impact on these levels.

**Methods:**

This retrospective study was conducted in an outpatient nephrology clinic and included data from 632 patients with CKD stages 1–5.

**Results:**

Serum phosphate was negatively associated with age (r = –0.08; p = 0.04) and estimated glomerular filtration rate (eGFR) (r = –0.23; p < 0.001). Women had significantly higher serum phosphate levels than men (3.6 mg/dL vs. 3.3 mg/dL; p = 0.01), an association confirmed in multivariate analysis. No significant sex differences were observed in serum calcium, parathyroid hormone (PTH), 25-hydroxyvitamin D, or the use of vitamin D supplements, calcium salts, or calcitriol.

**Conclusion:**

Even in the presence of CKD, women have higher serum phosphate (P) levels than men. This sex difference should be considered in the management of CKD–mineral and bone disorder (CKD-MBD).

## Introduction

Serum phosphate (P) is vital for human metabolism and has been linked to various clinical outcomes. Approximately 85% of total body P is stored in the bones, playing a key role in bone mineralization and formation^1^. Several studies have shown that increased serum P levels may be associated with a higher risk of kidney disease progression^2,3^, cardiovascular disease^4^, and mortality in patients with established chronic kidney disease^5^.

In the pre-menopausal period (before 45 years), studies have shown that there are no differences in P levels between the two sexes, whereas as women approach menopause and enter the postmenopausal period, a consistent increase in these levels may be observed^6^. This may also be associated with an increased risk of cardiovascular disease (CVD) in women after menopause^7^.

It is important to note that, despite the recognition of these differences, the current reference interval for phosphate has not been adapted, and such differences have not been investigated in the context of CKD, in which additional factors may exert a more pronounced impact on serum P levels^6^.

## Methods

The study protocol was approved by the local Research Ethics Committee (CAPPesq #45163715.4.0000.0068). Given the retrospective nature of the study, informed consent was waived. We retrospectively analyzed adult (≥18 years) outpatients followed for at least three months at the nephrology service of our institution (*Hospital das Clínicas da Faculdade de Medicina da Universidade de São Paulo*) between January 1, 2008, and December 31, 2017. Eligible patients had various kidney diseases—including glomerulopathies, hypertension, diabetes, and nephrolithiasis—and available data on serum phosphate and estimated glomerular filtration rate (eGFR). Individuals were divided according to the eGFR, obtained using the CKD-EPI equation (<45 or ≥45 mL/min/1.73 m^2^), and by age (18–65 and >65 years). Serum P was measured using a colorimetric assay, with a reference range of 2.5–4.5 mg/dL. Multivariable regression analysis was conducted with serum P as the dependent variable and total calcium, 25-hydroxyvitamin D, PTH, ancestry, eGFR, age, and sex as independent variables. Analyses were performed using R version 4.3, SPSS, and GraphPad Prism.

## Results

**Figure 1:**
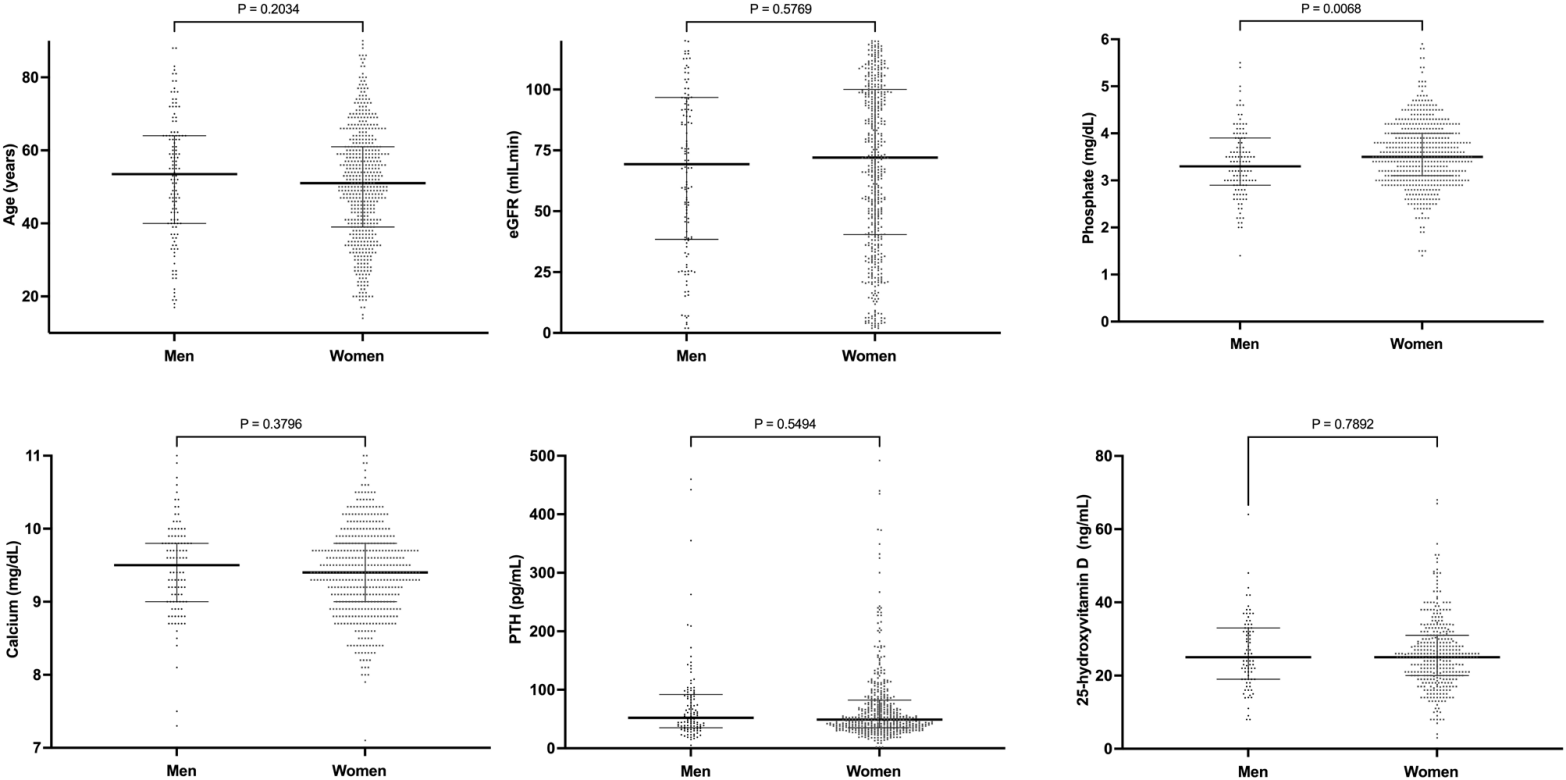
Comparison of clinical and laboratorial parameters between the sexes.

As shown in Table 1 and in Figure 1, our study population consisted of 632 patients, of whom 507 were women (80.2%). Most patients had CKD stage 1 (45.3%), followed by those with CKD stage 2 (31.3%). At all the disease stages, most patients were female. The median age of the cohort was 51 years (IQR: 39–62), with no statistically significant difference between the two sexes (Figure 1). Women with CKD stage 1 were slightly older than men, whereas the reverse occurred in CKD stage 4. We observed no differences in eGFR, although there was a tendency toward lower values in women with CKD stage 1.

**Table 1:** Markers of CKD studied by disease stage and sex

		Total Patients	CKD 1	CKD 2	CKD 3	CKD 4	CKD 5
M/W		125/507	57/229	43/155	31/139	14/50	6/17
Age, yrs	All	51 (39, 62)	43 (33, 52)	53 (42, 63)	57 (47, 69)	63 (53, 77)	51 (43, 59)
	Men	51 (39, 64)	36 (27, 50)^a^	53 (40, 64)	58 (48, 67)	76 (66, 79)^a^	50 (43, 60)
	Women	51 (40, 61)	45 (34, 53)	54 (42, 63)	59 (47, 70)	61 (49, 73)	50 (43, 59)
Ca, mg/dL	All	9.42 (9.0, 9.80)	9.30 (8.90, 9.70)	9.50 (9.20, 9.70)	9.50 (9.05, 9.90)	9.50 (9.00, 10.00)	9.50 (9.03, 10.10)
	Men	9.51 (9.2, 9.8)	9.45 (8.98, 9.82)	9.50 (9.30, 9.70)	9.50 (9.10, 9.80)	9.55 (9.43, 10.00)	9.50 (9.03, 9.98)
	Women	9.40 (9.0, 9.8)	9.30 (8.90, 9.65)	9.45 (9.10, 9.80)	9.50 (9.03, 9.98)	9.50 (8.95, 9.80)	9.50 (9.08, 10.13)
P, mg/dL	All	3.50 (3.00, 3.90)	3.40 (3.00, 3.80)	3.40 (3.00, 3.80)	3.49 (3.00, 3.95)	3.64 (3.10, 4.20)	4.72 (3.88, 5.65)
	Men	3.33 (2.93, 3.70)^a^	3.25 (2.93, 3.70)	3.30 (3.00, 3.50)	3.05 (2.68, 3.50)^a^	3.60 (3.15, 4.15)	5.05 (3.88, 5.58)
	Women	3.55 (3.10, 3.98)	3.40 (3.00, 3.90)	3.40 (3.00, 3.80)	3.50 (3.10, 4.00)	3.70 (3.10, 4.15)	4.65 (3.88, 5.65)
AP, UI/L	All	87 (58, 100)	66 (55, 90)	75 (60, 90)	69 (55, 91)	94 (73, 121)	91 (69, 120)
	Men	90 (57, 98)	101 (63, 128)	78 (59, 86)	61 (51, 74)	85 (76, 105)^a^	103 (90, 122)
	Women	86 (59, 101)	65 (55, 84)	75 (62, 101)	75 (59, 97)	97 (69, 122)	89 (68, 112)
PTH, pg/mL	All	76 (35, 75)	40 (30, 53)	43 (34, 58)	61 (42, 93)	107 (80, 172)	205 (134, 443)
	Men	76 (35, 74)	43 (31, 56)	41 (32, 61)	66 (43, 92)	80 (40, 113)^a^	373 (199, 541)
	Women	76 (35, 83)	40 (29, 53)	44 (37, 56)	60 (40, 92)	114 (87, 201)	170 (123, 389)
25(OH)D, ng/mL	All	26 (20, 31)	25 (19, 30)	26 (20, 30)	27 (22, 34)	26 (19, 33)	21 (17, 25)
	Men	26 (21, 32)	24 (20, 31)	25 (18, 29)	26 (23, 35)	30 (23, 35)	19 (17, 22)
	Women	26 (20, 31)	25 (19, 30)	26 (20, 31)	27 (21, 34)	25 (18, 32)	22 (17, 25)
eGFR	All	74 (49, 100)	106 (98, 116)	74 (68, 82)	47 (39, 54)	24 (21, 26)	7 (6, 14)
	Men	72 (47, 99)	110 (100, 122)	73 (68, 82)	47 (38, 53)	23 (20, 25)	7 (6, 7)
	Women	74 (50, 100)	105 (98, 114)	74 (68, 82)	47 (39, 54)	24 (21, 27)	11 (6, 14)
Dietary Vit D use, n (%)	All	80 (12.7)	34 (11.9)	19 (9.6)	17 (10)	7 (10.9)	3 (13)
	Men	10 (8.0)	7 (12.3)	2 (4.7)	1 (3.2)	0	0
	Women	70 (13.8)	27 (11.8)	17 (27)	16 (11.5)	7 (14)	3 (17.6)
Calcium carbonate use, n (%)	All	97 (15.3)	43 (15)	20 (10.1)	25 (14.7)	8 (12.5)	1 (4.3)
	Men	11 (8.8)	5 (8.8)	5 (11.6)	0	1 (7.1)	0
	Women	86 (16.9)	38 (16.6)	15 (34.2)	25 (18)	7 (14)	1 (5.9)
Calcitriol use, n (%)	All	14 (2.2)	7 (2.4)	0	3 (1.8)	3 (4.7)	1 (4.3)
	Men	2 (1.6)	2 (3.5)	0	0	0	0
	Women	12 (2.4)	5 (2.2)	0	3 (2.2)	3 (6)	1 (5.9)

Abbreviations – M: men; W: Women; Ca: calcium; P: phosphate; AP: alkaline phosphatase; PTH: parathyroid hormone; 25(OH)D: 25-hydroxyvitamin D; eGFR: estimated glomerular filtration rate.

Note – ^a^p < 0.05 vs. women at the same CKD stage.

Abbreviations – eGFR: estimated glomerular filtration rate; PTH: parathyroid hormone.

Note – Comparative analysis showing that women presented higher serum levels of phosphate, but no significant differences were observed regarding age, renal function, calcium, parathyroid hormone, or 25-hydroxyvitamin D.

Serum calcium and 25-hydroxyvitamin D were similar between sexes (overall and across CKD stages). No differences were identified in PTH and ALP; however, at CKD stage 4, women exhibited higher levels than men. Overall, women had significantly higher serum P levels than men. When we analyzed serum P according to CKD stages, we observed that this difference was statistically significant only in patients with CKD stage 3. Considering that, in the general population, increases in serum P are observed in postmenopausal women, we divided this group according to age and found no differences between them (median [25–75] vs. median [25–75] for those <50 vs. ≥50 years, respectively; p = 0.2637).

We additionally found no differences in the use of vitamin D supplements, calcium salts, or calcitriol.

**Figure 2:**
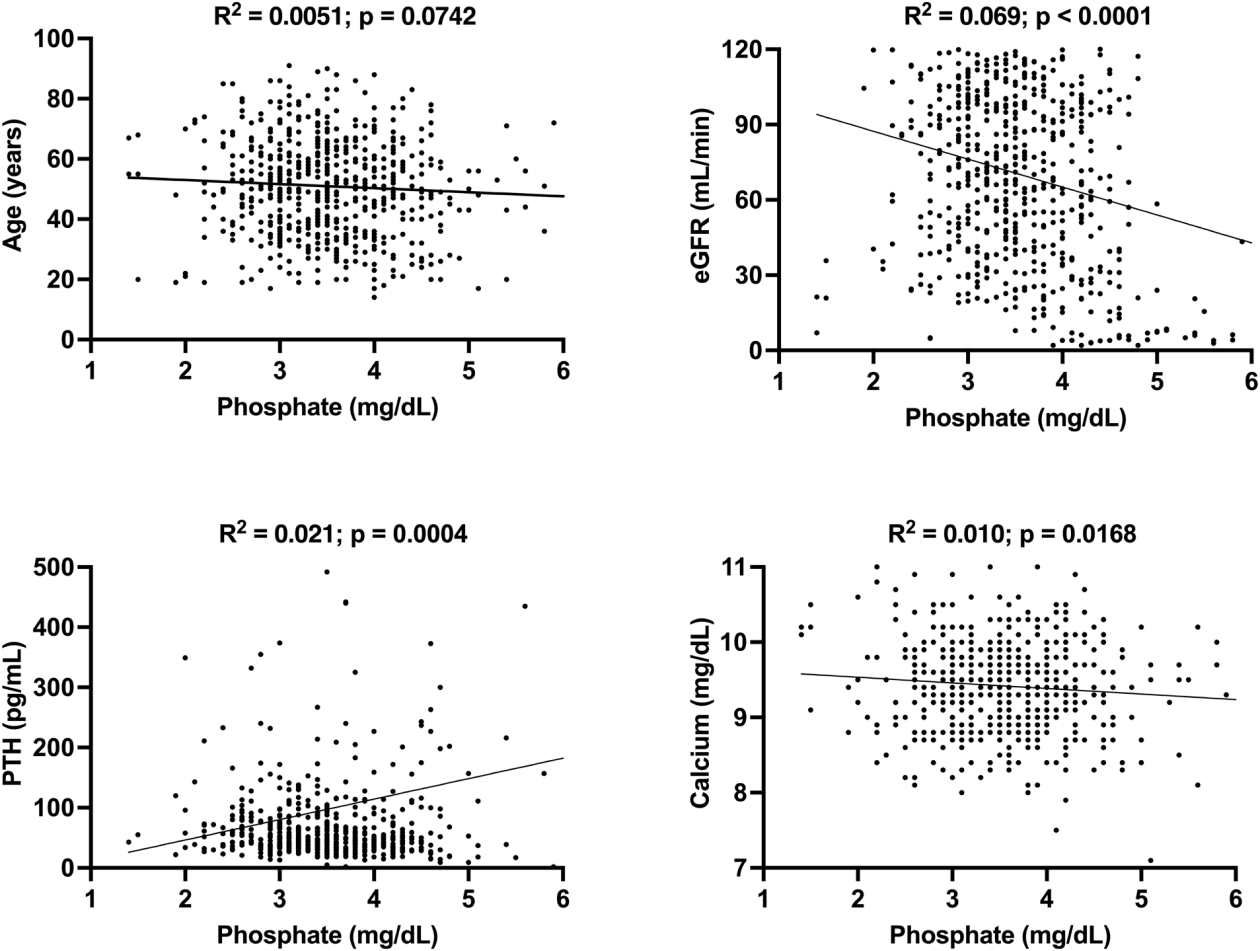
Correlation of clinical and laboratorial parameters with serum phosphate.

In our univariate analysis, we identified a significant association between serum phosphate and eGFR, PTH, and calcium levels (Figure 2). There was a tendency towards an association between serum P and age. As age increased, we observed a decrease in serum P levels. As expected, a decrease in eGFR was associated with an increase in serum P levels. Additionally, serum P was negatively associated with serum calcium. Regarding PTH, higher serum P levels were positively associated with higher serum PTH concentrations.

Note – Correlation analysis showing the association of phosphate with estimated glomerular filtration rate (eGFR), parathyroid hormone (PTH), and calcium.

In the multivariate regression analysis adjusted for serum calcium, 25-hydroxyvitamin D, PTH, and ethnicity, serum P was independently associated with eGFR, age, and sex (Table 2).

**Table 2:** Linear regression analysis of factors associated with serum phosphate

Model	Standardized β coefficients	p	Partial correlation
Constant		<0.001	
Total calcium	–0.084	0.093	–0.086
25(OH)D	–0.078	0.109	–0.082
PTH	–0.036	0.514	–0.034
White (race)	0.018	0.714	0.019
eGFR	–0.353	<0.001	–0.285
Age	–0.212	<0.001	–0.187
Sex	0.145	0.003	0.152

Note – Overall model: p < 0.001; adjusted R^2^ = 0.106.

## Discussion

Our analysis showed a significant association between serum P levels and sex in CKD patients. Our findings in this population were consistent with those reported in the general population.

The physiology underlying the changes observed in different parameters in relation to serum P in our results can be briefly explained. In the general population, with increasing age, a decrease in dietary intake of phosphate-rich foods, lower muscle mass, and reduced intestinal absorption of P are observed, all contributing to reduced serum P levels. Additionally, PTH levels increase with aging, which may enhance renal P excretion and consequently reduce serum P concentrations^8^. However, as eGFR decreases with the progression of CKD, the filtered load of P also decreases, thus causing its accumulation in the blood. High P levels bind to calcium and create calcium–P complexes, thereby lowering free calcium in the blood. This decrease in calcium levels stimulates the release of PTH in an attempt to increase calcium resorption from bone, decrease renal P reabsorption, and increase its excretion. In CKD, this process of P excretion is impaired due to reduced eGFR, therefore leading elevated PTH levels to indirectly increase serum P levels, and a correlation that is normally negative in the healthy population becomes positive. This is consistent with our findings, as serum P was positively associated with PTH and negatively associated with eGFR in our cohort.

The primary objective of this study was to assess whether the established sex differences in serum P levels are also present in CKD patients. As noted above, this phenomenon has been previously described in the general population, especially among postmenopausal women^6^. An experimental study demonstrated that estrogen downregulates the NaPi-IIa tubular cotransporter, thereby increasing renal P excretion^9^. Subsequently, another study suggested that this effect may be mediated by fibroblast growth factor 23 (FGF-23)^10^. In other words, estrogen appears to stimulate FGF-23 synthesis, which in turn inhibits NaPi-IIa expression, promoting phosphaturia. With the onset of menopause, estrogen levels decline markedly, which may contribute to the observed sex differences in serum P levels. However, CKD is typically accompanied by marked alterations in calcium, P, PTH, and FGF-23, raising the question of whether the sex-specific effect on serum P might be attenuated in these patients. Nonetheless, our findings confirm that this difference persists in CKD.

An important remaining question is whether elevated serum P directly contributes to the increased risk of cardiovascular disease and accelerated CKD progression observed in postmenopausal women. However, this hypothesis warrants further investigation in prospective studies. If this is the case, it will have an impact on how we consider treatment and monitoring in women with CKD-MBD compared with their male counterparts. It may be necessary to implement new reference guidelines that highlight sex differences, with emphasis on the relative serum P reference ranges.

This study was limited by the small sample size. Patient data were only collected from one clinic in our hospital, with a high proportion of female patients. Furthermore, in this CKD population, many younger women may have had amenorrhea, making it difficult to identify which individuals would physiologically behave as postmenopausal despite their chronological age. In addition, information on fasting status was unavailable, and serum P levels may have been obtained at varying times of the day across patients, thereby limiting the interpretation of the data^11,12^. Given the observational nature of the study, we believe the impact of our findings should be assessed in further studies with larger samples.

## Conclusion

In conclusion, our findings confirm that, even in the presence of CKD, women have higher serum P levels than men. This sex difference should be considered in the management of CKD-MBD. Furthermore, the question of whether women with CKD experience more cardiovascular adverse effects from elevated P compared to men warrants further investigation.

## Data Availability

The datasets generated and/or analyzed during the current study are available from the corresponding author upon reasonable request.
