# Supplementary material for: Sex-specific differences in serum phosphate persist in patients with CKD
Source: J Bras Nefrol. 2026 May 11;48(3):e20250345. doi: 10.1590/2175-8239-JBN-2025-0345en (PMC13160562; doi:10.1590/2175-8239-JBN-2025-0345en)
Supplement: Translation

# Translation: Diferenças entre os sexos nos níveis de fosfato sérico persistem em pacientes com DRC

**DOI:** 10.1590/2175-8239-JBN-2025-0345pt

**Authors:** Martina Schatz, Rafael Tavares, Luciene M dos Reis, Rosilene M. Elias, Rosa M. A. Moyses

**Affiliations:** 1 University of Edinburgh Medical School, Edinburgh, United Kingdom.; 2 Universidade de São Paulo, Faculdade de Medicina, Hospital das Clínicas, Serviço de Nefrologia, SP, Brasil.; 3 Universidade de São Paulo, Faculdade de Medicina, LIM 16 do Hospital das Clínicas, SP, Brasil.

**Keywords:** Insuficiência Renal Crônica, Distúrbio Mineral e Ósseo da Doença Renal Crônica, Fosfato

## Abstract

**Introdução:**

O fosfato desempenha um papel fundamental em diversos processos metabólicos, e diferenças específicas entre os sexos já foram identificadas em suas concentrações na população geral. No entanto, tais diferenças ainda não foram investigadas no contexto da DRC, no qual fatores adicionais podem exercer um impacto mais pronunciado sobre esses parâmetros.

**Métodos:**

Este estudo retrospectivo foi conduzido em um ambulatório de nefrologia e incluiu dados de 632 pacientes com DRC nos estágios 1–5.

**Resultados:**

O fosfato sérico apresentou associação negativa com a idade (r = –0,08; p = 0,04) e com a taxa de filtração glomerular estimada (TFGe) (r = –0,23; p < 0,001). As mulheres apresentaram níveis séricos de fosfato significativamente mais elevados do que os homens (3,6 mg/dL vs. 3,3 mg/dL; p = 0,01), resultado confirmado na análise multivariada. Não foram observadas diferenças significativas entre os sexos nos níveis séricos de cálcio, paratormônio (PTH), 25-hidroxivitamina D, nem no uso de suplementos de vitamina D, sais de cálcio ou calcitriol.

**Conclusão:**

Mesmo na presença de DRC, as mulheres apresentam níveis séricos de fosfato mais elevados do que os homens. Essa diferença entre os sexos deve ser considerada no manejo do distúrbio mineral e ósseo da DRC (DMO-DRC).

## Introdução

O fosfato (P) sérico é essencial para o metabolismo humano e tem sido associado a diversos desfechos clínicos. Aproximadamente 85% do fosfato total do organismo encontra-se nos ossos, desempenhando um papel fundamental na mineralização e na formação óssea^1^. Múltiplos estudos demonstraram que o aumento do P sérico pode estar associado a um maior risco de progressão da doença renal^2,3^, doença cardiovascular^4^ e mortalidade em pacientes com doença renal crônica já estabelecida^5^.

Na perimenopausa (antes dos 45 anos), estudos demonstraram não haver diferenças nos níveis de P entre os dois sexos; no entanto, à medida que as mulheres se aproximam da menopausa e entram no período pós-menopausa, observa-se um aumento consistente desses níveis^6^. Esse fenômeno também pode estar relacionado ao aumento do risco de doença cardiovascular (DCV) em mulheres após a menopausa^7^.

É importante destacar que, apesar do reconhecimento dessas diferenças, o intervalo de referência atual para o fosfato não foi adaptado, e tais diferenças ainda não foram investigadas no contexto da doença renal crônica (DRC), no qual fatores adicionais podem exercer um impacto mais pronunciado nesses parâmetros^6^.

## Métodos

O protocolo de estudo foi aprovado pelo Comitê de Ética (CAPPesq #45163715.4.0000.0068). Devido à natureza retrospectiva do trabalho, a obtenção de consentimento informado foi dispensada. Realizou-se uma análise retrospectiva de pacientes ambulatoriais adultos (≥18 anos) acompanhados por pelo menos três meses no serviço de nefrologia de nossa instituição (Hospital das Clínicas da Faculdade de Medicina da Universidade de São Paulo) entre 01/01/2008 e 31/12/2017. Os pacientes elegíveis apresentavam diversas doenças renais—incluindo glomerulopatias, hipertensão, diabetes e nefrolitíase—e possuíam dados disponíveis sobre P sérico e taxa de filtração glomerular estimada (TFGe). Os indivíduos foram divididos de acordo com a TFGe, obtida pela equação CKD-EPI (<45 ou ≥45 mL/min/1,73 m^2^), e pela idade (18–65 anos e >65 anos). O P sérico foi medido por meio de ensaio colorimétrico, com intervalo de referência de 2,5–4,5 mg/dL. A análise de regressão multivariada foi realizada com o P sérico como variável dependente e cálcio total, 25-hidroxivitamina D, PTH, ancestralidade, TFGe, idade e sexo como variáveis independentes. As análises foram executadas com os softwares R versão 4.3, SPSS e *GraphPad Prism*.

## Resultados

Conforme demonstrado na Tabela 1 e na Figura 1, nossa população de pacientes consistiu em 632 indivíduos, dos quais 507 eram mulheres (80,2%). A maioria dos pacientes apresentava DRC estágio 1 (45,3%), seguida pelo estágio 2 (31,3%). Em todos os estágios da doença, predominou o sexo feminino. A mediana de idade da coorte foi de 51 anos (IIQ: 39–62), sem diferença estatisticamente significativa entre os sexos (Figura 1). As mulheres com DRC estágio 1 eram ligeiramente mais velhas que os homens, ao passo que o oposto foi observado no estágio 4. Não foram identificadas diferenças significativas na TFGe, embora tenha sido notada uma tendência a valores mais baixos em mulheres com DRC estágio 1.

**Tabela 1:** Marcadores da DRC estudados por sexo e estágio da doença

		Total	DRC 1	DRC 2	DRC 3	DRC 4	DRC 5
H/M		125/507	57/229	43/155	31/139	14/50	6/17
Idade, anos	Todos	51 (39; 62)	43 (33; 52)	53 (42; 63)	57 (47; 69)	63 (53; 77)	51 (43; 59)
	Homens	51 (39; 64)	36 (27; 50)a	53 (40; 64)	58 (48; 67)	76 (66; 79)a	50 (43; 60)
	Mulheres	51 (40; 61)	45 (34; 53)	54 (42; 63)	59 (47; 70)	61 (49; 73)	50 (43; 59)
Ca, mg/dL	Todos	9,42 (9,0; 9,80)	9,30 (8,90; 9,70)	9,50 (9,20; 9,70)	9,50 (9,05; 9,90)	9,50 (9,00; 10,00)	9,50 (9,03; 10,10)
	Homens	9,51 (9,2; 9,8)	9,45 (8,98; 9,82)	9,50 (9,30; 9,70)	9,50 (9,10; 9,80)	9,55 (9,43; 10,00)	9,50 (9,03; 9,98)
	Mulheres	9,40 (9,0; 9,8)	9,30 (8,90; 9,65)	9,45 (9,10; 9,80)	9,50 (9,03; 9,98)	9,50 (8,95; 9,80)	9,50 (9,08; 10,13)
P, mg/dL	Todos	3,50 (3,00; 3,90)	3,40 (3,00; 3,80)	3,40 (3,00; 3,80)	3,49 (3,00; 3,95)	3,64 (3,10; 4,20)	4,72 (3,88; 5,65)
	Homens	3,33 (2,93; 3,70) a	3,25 (2,93; 3,70)	3,30 (3,00; 3,50)	3,05 (2,68; 3,50) a	3,60 (3,15; 4,15)	5,05 (3,88; 5,58)
	Mulheres	3,55 (3,10; 3,98)	3,40 (3,00; 3,90)	3,40 (3,00; 3,80)	3,50 (3,10; 4,00)	3,70 (3,10; 4,15)	4,65 (3,88; 5,65)
FA, UI/L	Todos	87 (58; 100)	66 (55; 90)	75 (60; 90)	69 (55; 91)	94 (73; 121)	91 (69; 120)
	Homens	90 (57; 98)	101 (63; 128)	78 (59; 86)	61 (51; 74)	85 (76; 105) a	103 (90; 122)
	Mulheres	86 (59; 101)	65 (55; 84)	75 (62; 101)	75 (59; 97)	97 (69; 122)	89 (68; 112)
PTH, pg/mL	Todos	76 (35; 75)	40 (30; 53)	43 (34; 58)	61 (42; 93)	107 (80; 172)	205 (134; 443)
	Homens	76 (35; 74)	43 (31; 56)	41 (32; 61)	66 (43; 92)	80 (40; 113) a	373 (199; 541)
	Mulheres	76 (35; 83)	40 (29; 53)	44 (37; 56)	60 (40; 92)	114 (87; 201)	170 (123; 389)
25(OH)D, ng/mL	Todos	26 (20; 31)	25 (19; 30)	26 (20; 30)	27 (22; 34)	26 (19; 33)	21 (17; 25)
	Homens	26 (21; 32)	24 (20; 31)	25 (18; 29)	26 (23; 35)	30 (23; 35)	19 (17; 22)
	Mulheres	26 (20; 31)	25 (19; 30)	26 (20; 31)	27 (21; 34)	25 (18; 32)	22 (17; 25)
TFGe	Todos	74 (49; 100)	106 (98; 116)	74 (68; 82)	47 (39; 54)	24 (21; 26)	7 (6; 14)
	Homens	72 (47; 99)	110 (100; 122)	73 (68; 82)	47 (38; 53)	23 (20; 25)	7 (6; 7)
	Mulheres	74 (50; 100)	105 (98; 114)	74 (68; 82)	47 (39; 54)	24 (21; 27)	11 (6; 14)
Uso de Vit D, n (%)	Todos	80 (12,7)	34 (11,9)	19 (9,6)	17 (10)	7 (10,9)	3 (13)
	Homens	10 (8,0)	7 (12,3)	2 (4,7)	1 (3,2)	0	0
	Mulheres	70 (13,8)	27 (11,8)	17 (27)	16 (11,5)	7 (14)	3 (17,6)
Uso de Carbonato de Cálcio, n (%)	Todos	97 (15,3)	43 (15)	20 (10,1)	25 (14,7)	8 (12,5)	1 (4,3)
	Homens	11 (8,8)	5 (8,8)	5 (11,6)	0	1 (7,1)	0
	Mulheres	86 (16,9)	38 (16,6)	15 (34,2)	25 (18)	7 (14)	1 (5,9)
Uso de Calcitriol, n (%)	Todos	14 (2,2)	7 (2,4)	0	3 (1,8)	3 (4,7)	1 (4,3)
	Homens	2 (1,6)	2 (3,5)	0	0	0	0
	Mulheres	12 (2,4)	5 (2,2)	0	3 (2,2)	3 (6)	1 (5,9)

Abreviaturas – H: homens; M: mulheres; Ca: cálcio; P: fosfato; FA: fosfatase alcalina; PTH: paratormônio; 25(OH)D = 25-hidroxivitamina D; TFGe: taxa de filtração glomerular estimada.

Nota – ^a^p < 0,05 vs. mulheres no mesmo estágio de DRC.

**Figura 1:** Comparação de parâmetros clínicos e laboratoriais entre os sexos.

Abreviaturas – TFGe: taxa de filtração glomerular estimada; PTH: paratormônio.

Nota – Análise comparativa demonstrando que mulheres apresentaram níveis séricos mais elevados de P; porém, não houve diferenças significativas em relação à idade, função renal, cálcio, paratormônio (PTH) ou 25-hidroxivitamina D.

O cálcio sérico e a 25-hidroxivitamina D foram semelhantes entre os sexos (tanto no grupo geral quanto ao longo dos estágios da DRC). Ainda que não tenham sido observadas diferenças significativas em PTH e fosfatase alcalina (FA), verificou-se que, no estágio 4 da doença, as mulheres apresentavam valores mais elevados do que os homens. De maneira geral, o grupo de mulheres apresentou níveis séricos de P significativamente mais elevados do que os homens. Ao analisar o P sérico de acordo com os estágios da DRC, observou-se que essa diferença foi estatisticamente significativa apenas nos pacientes com DRC estágio 3. Considerando que, na população geral, aumentos nos níveis de P sérico são observados em mulheres pós-menopáusicas, esse grupo foi estratificado por idade, e não foram identificadas diferenças entre as categorias (mediana [25–75] vs. mediana [25–75] para <50 anos vs. ≥50 anos, respectivamente; p = 0,2637).

Além disso, não foram encontradas diferenças relacionadas ao uso de suplementos de vitamina D, sais de cálcio ou calcitriol.

Na análise univariada, observou-se uma associação significativa entre P sérico e TFGe, níveis de PTH e níveis de cálcio (Figura 2). Houve uma tendência de associação entre o P sérico e a idade: à medida que a idade aumentava, observou-se uma redução nos níveis de P. Como esperado, a diminuição da TFGe esteve associada ao aumento desses níveis. Adicionalmente, o P sérico apresentou associação negativa com o cálcio sérico. Quanto ao PTH, valores mais elevados de P sérico estiveram positivamente associados a concentrações mais elevadas de PTH.

**Figura 2:** Correlação de parâmetros clínicos e laboratoriais com o P Sérico.

Nota – A análise de correlação mostra a associação do P sérico com a taxa de filtração glomerular estimada (TFGe), paratormônio (PTH) e cálcio.

Na análise de regressão multivariada, ajustada para cálcio sérico, 25-hidroxivitamina D, PTH e ancestralidade, o P sérico mostrou associação independente com TFGe, idade e sexo (Tabela 2).

**Tabela 2:** Análise de regressão linear dos fatores associados ao fosfato sérico

Modelo	Coeficientes β padronizados	p	Correlação parcial
Constante		<0,001	
Cálcio Total	–0,084	0,093	–0,086
25(OH)D	–0,078	0,109	–0,082
PTH	–0,036	0,514	–0,034
Branco	0,018	0,714	0,019
TFGe	–0,353	<0,001	–0,285
Idade	–0,212	<0,001	–0,187
Sexo	0,145	0,003	0,152

Nota – Modelo global p <0,001; R^2^ ajustado = 0,106.

## Discussão

Nossa análise demonstrou uma associação significativa entre os níveis séricos de P e o sexo em pacientes com DRC. Nossos achados nessa população foram consistentes com aqueles descritos na população geral. A fisiologia subjacente às alterações observadas em diferentes parâmetros em relação ao P sérico em nossos resultados pode ser brevemente explicada. Na população geral, com o aumento da idade, observa-se uma redução na ingestão de alimentos ricos em P, bem como na massa muscular e na absorção intestinal desse mineral, fatores que contribuem para a diminuição de seus níveis séricos. Além disso, os níveis de PTH aumentam com o envelhecimento, o que pode potencializar a excreção renal de P e, consequentemente, reduzir suas concentrações séricas^8^. No entanto, à medida que a TFGe diminui com a progressão da DRC, a carga filtrada de P também se reduz, levando ao acúmulo desse mineral no sangue. Concentrações elevadas de P se ligam ao cálcio, formando complexos de cálcio e P, o que reduz o cálcio livre no sangue. Essa diminuição dos níveis de cálcio estimula a liberação de PTH, na tentativa de aumentar a reabsorção óssea de cálcio, diminuir a reabsorção renal de P e aumentar sua excreção.

Na DRC, esse processo de excreção de P é prejudicado devido à redução da TFGe; assim, os níveis elevados de PTH acabam aumentando indiretamente o P sérico, transformando uma correlação que normalmente seria negativa na população saudável em uma correlação positiva. Esse padrão é consistente com nossos achados, já que o P sérico apresentou associação positiva com o PTH e associação negativa com a TFGe em nossa coorte.

O objetivo principal deste estudo foi avaliar se as diferenças entre os sexos no P sérico também estão presentes em pacientes com DRC. Como mencionado anteriormente, esse fenômeno já foi descrito na população geral, especialmente entre mulheres pós-menopáusicas^6^. Um estudo experimental demonstrou que o estrogênio regula negativamente o cotransportador tubular NaPi-IIa, aumentando, assim, a excreção renal de P^9^. Posteriormente, outro estudo sugeriu que esse efeito pode ser mediado pelo fator de crescimento de fibroblastos 23 (FGF-23)^10^. Em outras palavras, o estrogênio parece estimular a síntese de FGF-23, que, por sua vez, inibe a expressão de NaPi-IIa, promovendo a fosfatúria. Com o início da menopausa, os níveis de estrogênio diminuem de forma acentuada, o que pode contribuir para as diferenças entre os sexos observadas no P sérico. No entanto, a DRC é tipicamente acompanhada por alterações marcantes em cálcio, P, PTH e FGF-23, levantando a questão de se o efeito específico do sexo sobre o P sérico pode ser atenuado nesses pacientes. Apesar disso, nossos achados confirmaram que essa diferença persiste na DRC.

Uma questão importante que permanece é se o aumento do P sérico contribui diretamente para o maior risco de doença cardiovascular e para a progressão acelerada da DRC observados em mulheres pós-menopáusicas. Contudo, essa hipótese requer investigação adicional por meio de estudos prospectivos. Caso seja confirmada, exercerá impacto na forma como consideramos o tratamento e o acompanhamento de mulheres com DMO-DRC em comparação aos homens. Pode ser necessário implementar novas diretrizes de referência que evidenciem as diferenças entre os sexos, com ênfase nos intervalos de referência relativos de P sérico.

Este estudo apresenta limitações, incluindo o pequeno tamanho amostral. Os dados dos indivíduos foram coletados somente em uma clínica de nosso hospital, com uma alta proporção de pacientes do sexo feminino. Além disso, nesta população com DRC, muitas mulheres mais jovens podem ter apresentado amenorreia, dificultando a identificação de quais indivíduos se comportariam fisiologicamente como pós-menopáusicas, independentemente da idade cronológica. Adicionalmente, não havia informações sobre o estado de jejum, e os níveis séricos de P podem ter sido mensurados em diferentes horários do dia, o que limita a interpretação dos dados^11,12^. Por se tratar de um estudo observacional, acreditamos que o impacto de nossos achados deve ser avaliado em estudos futuros com amostras maiores.

## Conclusão

Em conclusão, nossos achados confirmam que, mesmo na presença de DRC, as mulheres apresentam níveis séricos de P mais elevados do que os homens. Essa diferença entre os sexos deve ser considerada no manejo do DMO-DRC. Além disso, a hipótese de que as mulheres com DRC apresentam maior ocorrência de efeitos cardiovasculares adversos devido ao aumento do P sérico em comparação aos homens merece investigação adicional.

## Data Availability

O conjunto de dados gerados e/ou analisados durante o presente estudo está disponível junto ao autor correspondente, mediante solicitação justificada.
